# Application of the market-ready NAVETTA electrodeposition chamber for controlled *in vitro* exposure with nano-scaled aerosols

**DOI:** 10.1016/j.csbj.2024.12.008

**Published:** 2024-12-17

**Authors:** Magdalena Weiss, Benjamin Punz, Jo Van Laer, An Jacobs, Sylvie Remy, Lisa Kleon, Vanessa Auer, Martin Himly, Sandra Verstraelen, Evelien Frijns

**Affiliations:** a Dept. Biosciences & Medical Biology, Paris Lodron University Salzburg (PLUS), Hellbrunnerstrasse 34, Salzburg 5020, Austria; b Environmental Intelligence Unit, Flemish Institute for Technological Research (VITO), Boeretang 200, Mol 2400, Belgium

**Keywords:** Aerosol, Air-liquid interface, Electrostatic deposition, Inhalation toxicology, *In vitro*, Hazard identification

## Abstract

Exposure of lung epithelia to aerosols is omnipresent. Chronic exposure to polluted air is a significant factor in the development of pulmonary diseases, which are among the top global causes of death, including COVID-19, chronic obstructive pulmonary disease, lung cancer, and tuberculosis. As efforts to prevent and treat lung diseases increase, the development of pulmonary drug delivery systems has become a major area of interest. In line with the ‘3 R’ principles (Reduce, Refine, and Replace animal testing), we developed an *in vitro* aerosol exposure system, termed NAVETTA, which was designed to replicate lung conditions most realistically. This system exposes air-liquid interface-cultured lung epithelial cells to a low, laminar airflow, enabling efficient aerosol deposition within an electric field. The aim of this study was to test instrumental performance on different aerosols, with a focus on precision, reproducibility, and cellular response. Deposition of sodium fluorescein droplets, pristine, and fluorescently labeled silica nanoparticles was homogenous and reproducible across the different instrument positions and over several runs, hence, the coefficient of variance for run-to-run and position-to-position was below 15 % using reference aerosols. To showcase NAVETTA’s versatile applicability, pristine silica nanoparticles and surface-functionalized fluorescently labeled silica nanoparticles were used. Various charging scenarios were studied, evidencing that deposition was enabled by and dependent on the applied electric field. Additional aerosol charging enhanced deposition compared to deposition achieved employing only the intrinsic charges of aerosol particles/droplets. In a second feasibility study two dry powder generators were tested for application with the NAVETTA system for testing deposition and cellular effects of nano-scale TiO_2_ aerosols. Cellular stress response was determined by interleukin-8 secretion, and viability post-exposure to TiO_2_ was monitored. Cells exhibited a trend to decreased viability and increased interleukin-8 secretion upon TiO_2_ deposition evidencing feasibility for application, however, more work is needed for optimizing reproducibility when using dry aerosol generators due to their discontinuous operation mode. Physiological conditions of 37°C and 98 % relative humidity within the NAVETTA resulted in 95 % viability over 24 h enabling longer-term exposure experiments. In summary, the market-ready NAVETTA presents a versatile exposure system for future *in vitro* pulmonary safety and efficacy studies by facilitating reliable and reproducible electrodeposition of various aerosols.

## Highlights

• The market-ready *in vitro* aerosol exposure chamber NAVETTA enables electrostatic enhanced deposition.
• Ability to use various aerosolizers to generate aerosols from liquid solutions, particle suspensions, or dry powder.
• Satisfying reproducibility across the four positions and several runs, coefficient of variance < 15 %.
• Four positions function as individual biological replicates for resource-efficient experiments.
• Safety testing under more realistic conditions for alveolar exposure.

## Graphical Abstract

**Figure:**
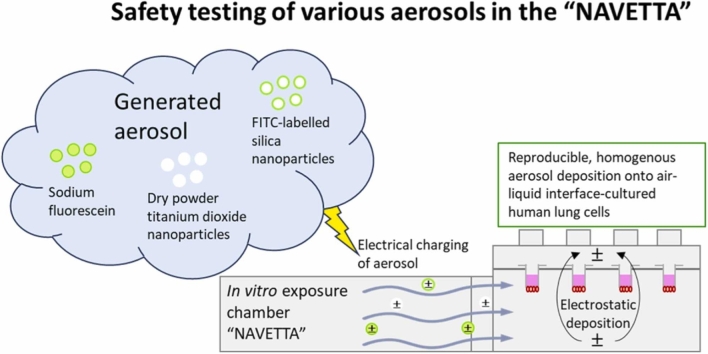

## 1 Introduction

Respiratory diseases represent a significant global health burden. In 2021, the World Health Organization listed COVID-19 (ranked 2nd), chronic obstructive pulmonary disease (ranked 4th), acute lower respiratory infections (pneumonia, ranked 5th), lung cancer (ranked 6th), and tuberculosis (ranked 10th) within the top ten leading causes of death worldwide [1]. Given the large and growing burden of respiratory diseases, the application of therapeutics also *via* the pulmonary route is gaining attention for targeted delivery of drugs [2]. In chemical toxicity screening in occupational settings and risk assessment of air pollution, a shift from *in vivo* towards *in vitro* assessment and *in silico* prediction methodologies has become imperative, following the ‘3 R’ principle (replacement, reduction and refinement of animal experiments) [3], [4], [5]. The directive 2010/63/EU of the European Parliament and Council underlines that the final goal is “*full replacement of procedures on live animals for scientific and educational purposes”*. However, it also states that for safety and efficacy regulatory testing procedures are necessary whose requirements can only be fulfilled by animal experiments [6]. In this context, the Safe-and-Sustainable-by-Design (SSbD) framework recommends a holistic approach to ensure that products, processes, and technologies are safe for humans and the environment, and sustainable throughout their life cycle [7]. The first step in the SSbD framework is dedicated to hazard identification, which is crucial for assessing the potential risks of new chemicals and materials [8]. Exposure to volatile materials can lead to respiratory sensitization, a significant public health concern due to its potential to cause chronic respiratory diseases and allergic reactions [9]. Accordingly in the United States, the Environmental Protection Agency announced that they will no longer support studies that use mammals to gauge the safety of chemicals by 2035 [10] and new medicines must not be tested in animals to receive Food and Drug Administration approval [11]. Novel processes utilizing new approach methodologies, propose the following workflow: first, *in silico* quantification of the expected lung-deposited dose is conducted using the Multiple-Path Particle Dosimetry model or more complex modeling such as computational fluid dynamics, physiologically-based pharmacokinetic modeling; this is followed by *in vitro* studies that, for the respiratory setting, use air-liquid interface (ALI) exposure systems to test the effects of the predicted dose [12]. To date, however, no *in vitro* models have been accepted by regulatory agencies as a complete replacement for inhalation testing in animals [13]. An overview of existing exposure systems (research-based and commercial) has been given by several groups [14], [15], [16], extending even to compact, robust, and mobile perpendicular flow exposure systems [17], [18]. Another previously published system [19], the NAVETTA flatbed ALI exposure chamber, offers the following unique characteristics and has meanwhile been developed towards market readiness: (i) inverted transwell set-up (cells are grown on the outside of the transwell membrane); (ii) low laminar lateral flow passing by the cell surface to mimic more realistic human lung cell exposure of the alveolar region; (iii) increased deposition rates by employment of an electrical field (EF); (iv) internal conditioning (humidification and temperature control) of aerosol flow to optimize cell viability for longer-term exposure times (up to 24 h) with > 95 % viability after clean air (CA) exposure of A549, the most commonly used cell line for human lung *in vitro* models having potential as an indicator system for *in vivo* studies of acute lung toxicity according to OECD Test Guideline 433 [20]. In that study, cell viability and immune responses after exposure to CuO nanoparticles (NP) had been reported [19]. The NAVETTA was further optimized in terms of customer-related issues (*e.g.* cleaning-friendly material, geometry, deposition variance) and will soon be commercially available. The aim of this study was to test instrumental performance, with a focus on precision, reproducibility, and feasibility for versatile applications on a set of different aerosols. To demonstrate proof-of-concept for instrumental optimization, *i.e.* deposition precision and reproducibility across four NAVETTA positions and over several repetitions, fluorescein droplets and fluorescent silica NP were aerosolized from aqueous solution and suspension, respectively, were employed. Longer-term survival for application in exposure experiments up to 24 h was demonstrated for human alveolar epithelial A549 cells. In feasibility studies the versatility of the NAVETTA for different operational modes and materials was tested: (i) effectiveness of the electrostatic deposition, (ii) deposition of differently functionalized, hence differently charged, silica NP under different EF conditions; (iii) dry powder aerosolization for exposure of cells to TiO_2_ NP. Here the system was applied for testing the pro-inflammatory potential and post-exposure viability of cells exposed to dry aerosol to verify previous data obtained from *in vivo* [21] experimentation using this novel *in vitro* platform employing ALI-cultured human lung A549 cells at a most realistic exposure scenario for inhalation (immuno)toxicology.

## 2 Materials and methods

### 2.1 The NAVETTA workplace

#### 2.1.1 The architecture of the exposure chamber

The NAVETTA was further developed towards market readiness by the Paris Lodron University of Salzburg (PLUS) and the Flemish Institute for Technological Research (VITO) based on their first prototype as described by Frijns et al. [19]. Patents were granted (European Unitary patent: EP3545302B1; Chinese patent: CN110140052B; US patent: US11598765B2), entitled “Flatbed air-liquid interface exposure module and methods”. The market-ready NAVETTA prototype was produced by the company VITROCELL Systems GmbH (Waldkirch, Germany) and is essentially made of stainless steel and non-conductive plastic. The NAVETTA consists of two units. The humidification unit holds an internal open water bath containing 91 mL of Millipore water. The cell unit includes a sample holder designed to hold four transwells in a hanging position. The water circuit of two external water baths (Huber KISS 104 A, Kältemaschinenbau AG, Offenburg, Germany) helps to warm up the NAVETTA to 37°C to create optimal conditions for the cells. To reach > 36°C at the temperature and humidity sensor (VITROCELL Systems GmbH) sitting next to the cells in the center of the NAVETTA, the water baths supplying the humidification and cell units are set to 38.5°C and 39°C, respectively. The temperature difference between the humidification and cell units is necessary to avoid condensation of the aerosol in the cell unit and compensates well for the thermal energy loss in the tubings. The humidity inside the chamber therefore stays > 98 % relative humidity (rH). The cell unit consists of a sample holder carrying a series of four transwells seeded with cells in inverted setup described in detail below. For the electrostatic deposition, an EF is generated within the cell unit. The lid carries four electrodes which protrude into the cell culture medium (CCM) of the transwells, the bottom of the unit consists of a grounded counter electrode to build up an EF. This achieves an enhanced electrostatic deposition. Each electrode can be controlled individually by the high-voltage power supply (Enhanced Deposition Controller, VITROCELL Systems GmbH). The polarity of the electrodes can be adjusted according to the experimental requirements.

#### 2.1.2 The experimental workflow

**Fig. 1:**
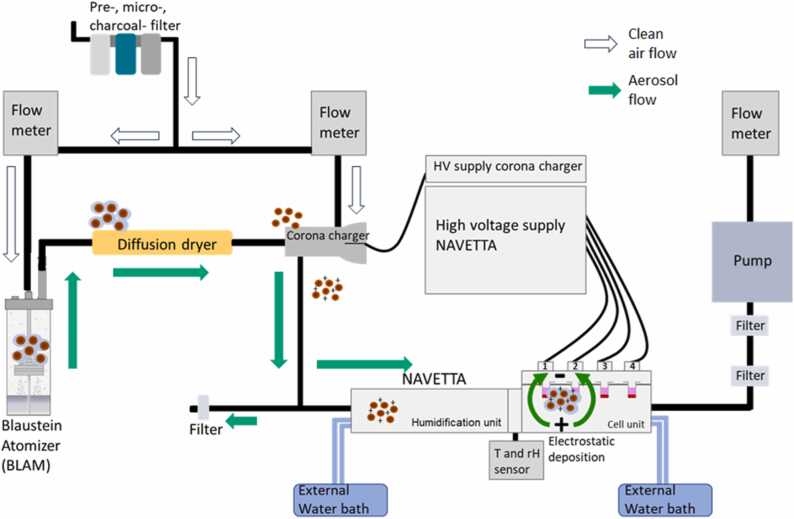
Schematic overview of the NAVETTA system. The scheme depicts the aerosol flow (green arrows) generated from clean air (white arrows) inside the Blaustein Atomizer (BLAM) and several relevant instrument components Flow meter, Filtering systems, Diffusion dryer, Corona charger, NAVETTA unit, Water baths, High-voltage (HV) power supply, relative humidity (rH) & temperature (T) sensors, and Pump of the NAVETTA setup. Optionally, the system was run using a collision-type atomizer or dry powder generator PowderX or PreciseInhale®. Experiments can be performed with and without a diffusion dryer. Further details (incl. photographs) can be found at https://doi.org/10.5281/zenodo.13862979.

The potential risk of aerosol exposure necessitates working under a fume hood and preferably within a biosafety level 2 laboratory, to allow for application of biological aerosols as well as to the use of primary cells and other advanced more complex cellular models. For air purification, a pre-filter, micro-filter, and activated carbon-filter (Riegler, Bad Urach, Germany) were employed for the compressed air. Three flow meters (Analyt-MTC Messtechnik GmbH, Mülheim, Germany) were used to regulate air flows in liter per minute (Lpm) for the aerosolization process, the corona charger (CC), and the low laminar air flow through the NAVETTA (Fig. 1). Fresh CCM with a volume of 200 µl was added into the upper compartment of the transwells before they were placed into the sample holder of the NAVETTA cell unit. Depending on the experiment, aerosol was generated in the Blaustein Atomizer (BLAM) (CH technologies, Westwood, NJ, USA), collision-type atomizer (model ATM 220, Topas, Dresden, Germany), the PreciseInhale® (PI) system (Inhalation Sciences, Huddinge, Sweden), and the PowderX (PX) system (VITROCELL Systems GmbH). If needed a diffusion dryer filled with silica gel (DRIERITE, Gel Chameleon from VWR, Leuven, Belgium) could be used drying the particles before charging them. In the CC (TSI, Shoreview, Minnesota, USA), clean air (CA) was ionized and mixed with the sample air at a ratio of 80 % sample air with 20 % ionized air. A THQ high-voltage power supply (Iseg, Radeberg, Germany) provided the necessary high-voltage for the CC set to ± 2.5 kV for each experiment. A vacuum pump (N480 LABOPORT KNF, Schenkon, Switzerland) positioned at the end of the cell unit (controlled by a flow meter) generated the air flow for the laminar flow inside the NAVETTA. Two filters (HEPA-CAP Whatman from Sigma, VITROCELL Systems GmbH) were used to prevent contamination of the flow meter regulating the air flow through the NAVETTA. The polarity of the electrodes on the lid of the cell unit can be controlled using a high-voltage supply (6 kV, 1.5 mA, model THQ T2070006EPU, ISEG Spezialelektronik GmbH, Radeberg, Germany) set at ± 1 kV for each position and experiment. The waste air was then extracted by the fume hood.

### 2.2 Cell culture

For the fluorescein aerosols and dry TiO_2_ NP, the human alveolar epithelial type 2-like A549 cell line was obtained from American Type Culture Collection (ATCC number: CCL-185, 80 passages, Manassas, USA), which was originally derived from a lung carcinomatous tissue from a 58-year-old Caucasian male. A549 cells were grown in T-75 culture flasks and routinely maintained in Dulbecco’s Modified Eagle Medium (DMEM) 1x with GlutaMAX™-1 (Brand Gibco, Thermo Fisher Scientific) supplemented with 10 % non-heat inactivated fetal bovine serum (FBS, Tico, Amstelveen, The Netherlands) at 37°C under 5 % carbon dioxide (CO_2_). Before reaching 70–80 % confluence, cells were subcultured using (0.05 %) Trypsin-EDTA solution (Brand Gibco, Thermo Fisher Scientific). Medium was refreshed every 2 days and cells were subcultured every 3 (9 ×10^5^ cells in 20 mL CCM) or 4 days (4.5 ×10^5^ cells in 20 mL CCM). Cells from the work cell bank were passaged at least 2 times before use in experiments and no more than 20 times in total. A549 cells were negative for mycoplasma.

Silica NP deposition experiments were performed using the reporter cell line A549 IL8-LUC, which was generated as previously described [22]. Cells were cultured and split following standard cell culture procedures in RPMI-1640 medium (Sigma, St. Louis, MO, USA) supplemented with 10 % non-inactivated FBS (Catus Biotech GmbH, Tutzing, Germany), 1 % glutamine (Sigma), 1 % penicillin/streptomycin (Sigma), Dulbecco's phosphate-buffered saline (PBS, Sigma) and 10 mM HEPES buffer (Sigma).

To prepare inverted ALI cultures (to be inserted into the cell unit of the NAVETTA), ThinCert® transparent transwells with a pore size of 0.4 µm and a surface area of 0.336 cm^2^ (24-well plate format) (Greiner Bio-One, Kremsmünster, Austria) were flipped upside down and placed in a Petri dish. Cells were detached using 1x Trypsin-EDTA (Sigma) and counted. A cell suspension with 1 × 10^6^ cells/mL was prepared, and 50 µL of this suspension, containing 50,000 cells, was placed on top of the transwell membrane (outer side). The Petri dish was closed and incubated for 2–2.5 h at 37°C and 5 % CO_2_. After incubation, the cell suspension droplet was removed, and the transwells were placed in a 24-well plate in a hanging position. The wells of the plate were filled with 800 µL of medium, and 100 µL of medium was added to the transwells. The cells were then incubated for 72 h at 37°C and 5 % CO_2_.

### 2.3 Cell viability

Cell survival inside the NAVETTA under operating conditions (CA flow rate through NAVETTA 0.2 Lpm) was tested up to 24 h by flow cytometry measurements using the Fixable Viability Dye eFluor506 (eBioscience, Waltham, MA, USA). This was confirmed using MTT assay (Merck, Darmstadt, Germany) for CA flow rate through NAVETTA at 0.2 Lpm and 0.3 Lpm. Furthermore, a MTT assay to address cell viability post-exposure with TiO_2_, as control for the pro-inflammatory readouts was performed.

For flow cytometry-based viability assessment the transwells were placed in a 24-well plate, hanging in empty wells and the apical applied CCM was removed. 300 µl Trypsin-EDTA was added basolateral and incubated for 5 minutes (min) (37°C, 5 % CO_2_). Afterwards, the plate was gently tapped and 300 µl CCM was added to inactivate Trypsin-EDTA. Transwells were then lifted out and the remaining attached cells were carefully resuspended with the medium-trypsin mixture. The cells were then collected and stained with Fixable Viability Dye eFluor506 (eBioscience, Waltham, MA, USA) and acquired using the flow cytometer Canto II flow cytometer (BD Biosciences, New Jersey, USA). The data thus obtained were analyzed using the FCSalyzer v.0.9.22-alpha software. The data are presented in relation to the live (24 h incubator)- and dead (cells incubated at 90°C for 1 min) controls, which has been obtained by excluding potential cell debris and doublets. The gating strategy shows the live/dead separation via applying a quadrant separation, which was aligned by assessing live and dead controls respectively by excluding cell debris, NP, and doublets. The gating strategy is shown at https://doi.org/10.5281/zenodo.13862979.

For assaying cell viability inside the NAVETTA under operating conditions and post-exposure with TiO_2_ NP from dry state, the MTT assay, determining the conversion of 3-(4,5-dimethylthiazol-2-yl)-2,5-diphenyltetrazolium bromide tetrazolium salt into its reduced formazan form, was performed. A MTT stock was prepared in Dulbecco’s PBS (Thermo Fisher Scientific) at a concentration of 5 mg/mL. The MTT substrate is prepared in CCM at a final concentration of 1 mg/mL, 300 µl is added basolateral and incubated for 2 h at 37°C and 5 % CO_2_. The formazan product of the MTT tetrazolium accumulates as an insoluble precipitate inside cells as well as being deposited near the cell surface and in the medium. The formazan was solubilized prior to recording absorbance readings by adding 500 µL isopropanol (Merck) basolateral and incubation for 2 h, shaking at room temperature. The quantity of formazan (presumably directly proportional to the number of viable cells) was measured by recording changes using a multi-mode microplate reader in absorbance mode (570 nm; Clariostar, BMG Labtech Ortenberg, Germany). Cell viability was expressed as the percentage of relative absorbance of treated cells relative to the control cells.

### 2.4 Scanning mobility particle sizer (SMPS)

The electrical mobility aerosol particle size distribution of the atomized fluorescein particles using the collision-type atomizer (model ATM 220), and the generated TiO_2_ nanoaerosol (PI, PX) was measured using a scanning mobility particle sizer (SMPS, model 3938L76, TSI Inc., Shoreview, MN, USA) that incorporates both a dynamic mobility analyzer (DMA) with aerosol neutralizer (Kr-85 370 MBq) and a butanol-based condensation particle counter (CPC) operating at a sample flow of 0.3 Lpm measuring in the size range of roughly 15–600 nm. Measurement of size distribution of sodium fluorescein and PBS aerosol generated with the BLAM was performed with SMPS from GRIMM Model 5.400, 55-U (DURAG group, Hamburg, Germany). A dynamic mobility analyzer (L-DMA) and a butanol-based CPC operating at a sample flow of 0.3 Lpm measuring in the size range of roughly 10 nm - 1 µm. Aerosol was neutralized in the DMA-based on the radioactive decay of ^241^Am (nominal activity 3.7 MBq).

### 2.5 Fluorescein aerosol generation from aqueous solution and deposition measurement

To confirm that the technical adjustments did not affect the working principle of the NAVETTA, a proof-of-concept study was performed using an aqueous solution of fluorescein sodium salt (Sigma Aldrich, Overijse, Belgium). Fluorescent aerosols were generated using a collision-type atomizer (model ATM 220, Topas, Dresden, Germany) containing a 50 mL 0.1 % fluorescein suspension and HEPA-filtered laboratory compressed air (5 bar). The obtained aerosol (4 Lpm) was dried using a diffusion dryer (model 3062, TSI, Shoreview, USA) resulting in salt crystals with a mode of roughly 100 nm diameter. A second air flow of 1 Lpm laboratory compressed air passed through a carbon- and HEPA-filter (HEPA/C) and an ionizer (corona jet charger, operated at +2.5 kV). Both flow streams were merged in a mixing chamber where particles in the aerosol flow mixed with the positive ions carried by the filtered CA. Using a T-split junction, 0.3 Lpm of the charged aerosol flow entered the NAVETTA using a pump connected to a mass flow controller (MFC) at NAVETTA’s outlet, while the remaining flow was led into the exhaust of the fume hood. Hanging inserts with A549 ALI-cultured cells were positioned inside the NAVETTA and nourished with 200 µl CCM pipetted inside the insert, the studs were connected to HV source (6 kV, 1.5 mA, model THQ T2070006EPU) set at −1 kV for each position. Cells were exposed for 15 min to the positively charged fluorescein aerosol. After exposure, inserts were removed and positioned in a 24-well plate with 200 µL distilled water. Subsequently, the inserts were removed from the well plate, and the fluorescence was measured in the well plate with a fluorometer (Clariostar, BMG Labtech, Ortenberg, Germany). Results were compared with a calibration curve.

### 2.6 Fluorescent silica NP-containing aerosols from PBS suspension

For aerosolization of silica NP (HIQ-Nano, Arnesano, Italy), a low flow, 4-jet nozzle BLAM was used. This atomizer operates on the principle of a collision atomizer, except for generating a very high aerosol concentration at low air flow rates (0.4–4 Lpm), making it particularly gentle. Additionally, droplets are sprayed downward and must pursue a 180° turn to reach the outlet at the top of the jar. This process results in a narrow size range of droplets, as larger droplets are unable to perform the U-turn and either adhere to the jar wall or fall back into the liquid reservoir (Fig. 1). The aerosolization experiments were operated from suspensions of 0.1 mg/mL NP in PBS. The NAVETTA system was used without a diffusion dryer to minimize sample loss. For similar reasons, a flow rate 0.7 Lpm was used for aerosol generation with the BLAM (2 mL of the sample suspension filled into the reservoir jar). Optionally, aerosol charging using a CC was applied; in such cases 0.175 Lpm ionized air was mixed into the stream (5:1 mixture). A549 cells were prepared following the inverted ALI seeding protocol. Transwells were inserted into the cell unit of the NAVETTA, re-filled with 200 µL CCM, and exposed to the aerosol flow at 0.2 Lpm for 30 min. After the exposure, cells were placed in an empty 24-well plate incubating for 3 h to facilitate uptake of deposited NP into the cells (37°C, 5 % CO_2_). The apical applied CCM was removed. 300 µl Trypsin-EDTA was added to the basolateral compartment and incubated for 5 min (37°C, 5 % CO_2_). Afterwards the plate was gently tapped and 300 µl CCM were added to inactivate Trypsin-EDTA. Transwells were then lifted out and the remaining attached cells were carefully resuspended with the medium-trypsin mixture. The cells were then collected and fixed by adding 4 % p-formaldehyde (Sigma) solution in PBS, before sample acquisition using the flow cytometer Canto II flow cytometer (BD Biosciences). The data obtained were analyzed using the FCSalyzer v.0.9.22-alpha software and are presented by comparing the Mean Fluorescent Intensity (MFI) of the fluorescence channel values of exposed to the values of unexposed transwells. The gating strategy presented results in mean fluorescence intensity (MFI), and this has been obtained by excluding the cell debris, NP, and doublets as shown at https://doi.org/10.5281/zenodo.13862979.

### 2.7 Titanium dioxide aerosol generation from dry powder and pro-inflammatory response determination

#### 2.7.1 Material and physicochemical characterization

For the TiO_2_ NP (nanoTiO_2_) case study, the particles (TiO_2_-NM105-JRCNM01005) were obtained from JRC Nanomaterials Repository (DG-JRC, Ispra, Italy). The particle diameter is 25–30 nm with an anatase:rutile ratio of 85:15. Detailed physicochemical characterization data of the material is available in Rasmussen et al., 2014 [23]. The hydrodynamic size obtained by dynamic light scattering was previously reported to be 153 ± 5.3 nm (upon 20 min sonication at 40 % amplitude in 10 mM HNO_3_), the reported zeta potential was 11.1 ± 0.7 mV, and the reported specific surface area, determined by Brunauer-Emmett-Teller, was ∼51 m^2^/g.

#### 2.7.2 Endotoxin monitoring

Detection and quantification of Gram negative bacterial endotoxin contamination on NP is an essential step in (immune)safety assessment of NMs in order to discriminate between effects resulting from endotoxin and intrinsic effects of the NP themselves [24], [25]. One of the most common endotoxin assays is the FDA-approved *Limulus Amebocyte* Lysate (LAL) assay, which is often considered as the standard method for endotoxin detection. The Kinetic Chromogenic LAL assay was used according to manufacturer’s instructions (LAL Kinetic-QCL™ Kinetic Chromogenic LAL Assay, including LAL Reagent, Control Standard Endotoxin (*E. coli* O55:B5), and LAL Reagent Water; cat.no. 50–650U; Lonza; Basel, Switzerland) using a synthetic substrate which is converted by the clotting enzyme to the colored compound p-nitroaniline, whose absorbance can be measured photometrically at 405 nm using an incubating temperature-controlled microplate reader.

#### 2.7.3 Dry powder aerosolization

Aerosols were generated from dry TiO_2_ powder using (i) the PI system as well as the (ii) PX system as shown in Fig. 1. A small amount of powder (1 mg) was loaded into the powder chamber, and an aerosol was generated (i) in the 300 mL holding chamber of the PI system using compressed air (100 bar), causing rapid de-agglomeration of the powder agglomerates, and (ii) in the ∼2500 mL holding chamber of the PX system using compressed air (5 bar), causing rapid de-agglomeration of the powder agglomerates, respectively. The aerosol was extracted from the holding chamber at a flow rate of 120 milliliter per minute (mL/min), in (i) also passed a real-time dust monitor (Casella, CEL-712, Kempston, Bedford, UK) to determine the generated aerosol concentration, and thereafter, it was guided through the NAVETTA exposure system with the 4 hanging cell inserts in place. To increase the deposition efficiency of the negatively charged TiO_2_ powder (-13.7 V) onto the cell surface, electrostatic precipitation was applied by connecting each of the four electrodes above the insert positions to a high-voltage unit set at + 1 kV. The aerosol exposure time for each generation cycle was 5 min, and aerosol flow rate through the NAVETTA was 0.12 Lpm.

#### 2.7.4 Titanium dioxide determination

For analysis of nanoTiO_2_ in/on the cells, the membrane with cells was removed from the insert with a scalpel blade, immediately after exposure and stored in a 15 mL tube at −20°C. For chemical analysis, the filter was placed into a conical digestion tube. Subsequently, 0.375 mL ultrapure hydrochloric acid (Thermo Fisher Scientific, Waltham, USA), 0.125 mL ultrapure nitric acid (Fisher Chemical), and 0.2 mL ultrapure hydrofluoric acid (Merck) were added. The digestion tubes were placed in a heating block during 120 min at a temperature of 105 °C. After cooling to room temperature, the tubes were filled up to a volume of 10 mL with water (Milli Q, Millipore) and placed in an ultrasonic bath for 60 min. The supernatant was then used for measuring the titanium content. The measurements were carried out with a high resolution inductively coupled-mass spectrometer (Element2, Thermo Fisher Scientific). The instrument was calibrated with standards ranging from 0 to 200 μg/L titanium (Labkings, The Netherlands) and verified with quality control standards ranging from 0.1 to 10 μg/L titanium (CPI International, USA). The titanium was measured on mass (*m/z*) 47, 48, and 49 in medium and high-resolution mode. The titanium result measured on *m/z* 47 in medium resolution mode was used for reporting.

#### 2.7.5 Pro-inflammatory readout

After exposure, cells were incubated by placing the inserts in a new sterile 24-well plate with fresh 200 µl CCM added apically, allowing recovery in a humidified incubator. During each run, three replicate inserts with A549 cells were treated in parallel in the NAVETTA (position 1, 2, and 4). Directly after exposure, one sample (one membrane with cells) was taken for chemical analysis (position 4). Hereafter, the 2 remaining inserts of position 1 and 2 were further incubated for 19–24 h for additional assessment of post-exposure cell viability and cytokine secretion. Three biologically independent runs, using different cell passages, were performed for each exposure condition. After exposure, the NAVETTA exposure module and all tubings were thoroughly rinsed with 70 % ethanol. After 19–24 h post-exposure, 200 μL of the apical medium was collected in a 24-well plate and stored at −80°C until use. For the measurement of the pro-inflammatory marker interleukin (IL)-8 cyto-/chemokine, a V-PLEX assay was performed according to manufacturer’s protocol (Meso Scale Discovery, Inc., Rockville, Maryland, USA). A549 cells were exposed to 20 µg/mL lipopolysaccharide (LPS) as positive control for cytokine secretion. This assay is an electrochemiluminescent detection method and is a highly sensitive multiplex enzyme-linked immunosorbent assay. Calibration curves were used to calculate the cytokine concentrations, expressed in pg/mL. Briefly, the V-PLEX plate was washed using 150 μL of wash buffer per well. Then, the calibrators were added, as well as the prepared samples. The plate was sealed and incubated for 2 h at room temperature. An additional washing step was performed 3 times, followed by the addition of 25 μL of the detection antibody solution to each well. The plate was incubated at room temperature for 2 h on a plate shaker. Following this, another washing step was performed 3 times. Subsequently, 150 μL of the read buffer T was added to each well, and the plate was analyzed on the MSD instrument. The MSD software calculates the protein concentrations in each sample based on the calibrators that were incubated alongside the samples.

## 3 Results

### 3.1 Aerosols within the respirable size range are generated from liquid and dry state

**Fig. 2:**
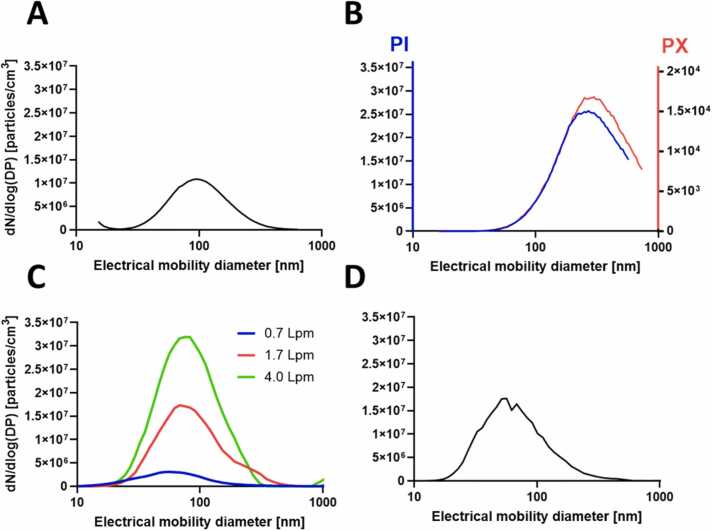
Particle size distributions of generated aerosols measured by SMPS. A. Fluorescein (5 mg/mL) droplets aerosolized by collision-type atomizer (model ATM 220); B. NanoTiO_2_ aerosol measured after one generation cycle of 1 mg using the PreciseInhale® (PI) system in blue and the PowderX (PX) system in red; C. Fluorescein (5 mg/mL) droplets aerosolized by BLAM using different flow rates 0.7, 1.7, 4.0 Lpm with diffusion dryer; D. PBS aerosolized by BLAM without using the diffusion dryer at 0.7 Lpm airflow.

Fluorescein droplets generated with the collision-type atomizer model ATM 220 were used to determine the spatial variability of the deposited dose across the four positions and for the NAVETTA exposure system in total. For the latter particles/droplets deriving from all sources will be uniformly termed particles to ease terminology. The aerosol particle size distribution of the generated fluorescein, nanoTiO_2_, and PBS is shown in Fig. 2. Fluorescein particle (Fig. 2A) mode was about 96 nm and particle mean 114 nm. The total particle number concentration was 6 * 10^6^ particles per cm^3^. The size distributions of fluorescein particles generated with the BLAM at different airflow rates are shown in Fig. 2C. An increase in airflow rate resulted in an increase in particle concentration. Particle mean diameter of aerosol generated at 0.7 Lpm was 70 nm, the particle mode was 57 nm. Particles generated at a 1.7 Lpm airflow rate had a particle mean of 98 nm and a particle mode of 68 nm. The highest airflow rate that can be used with the low flow BLAM (*i.e.* 4.0 Lpm) generated particles with a mean diameter of 93 nm and a particle mode of 78 nm. Dry powder aerosolization was performed using two different systems. The PI-generated TiO_2_ (Fig. 2B) aerosol showed a particle mode of 269 nm and mean diameter of 276 nm and the TiO_2_ aerosol generated by PX showed a particle mode of 300 nm and a mean diameter of 319 nm. The difference in concentration between the two devices can be explained by the larger holding chamber (2500 mL) of the PX generator compared to the PI holding chamber (300 mL). The mean TiO_2_ particle size generated by PX is slightly bigger than the one generated by PI probably due to the lower aerosolization pressure resulting in less deagglomeration of the initial TiO_2_ powder. PBS was the carrier solution of silica NP. The SMPS measurements were performed without the diffusion dryer. The size distribution of PBS aerosolized with the BLAM is shown in Fig. 2D. Particle mean was 75 nm and particle mode was 57 nm.

### 3.2 A549 cells show satisfying survival inside NAVETTA under operating conditions when tested up to 24 h

**Fig. 3:**
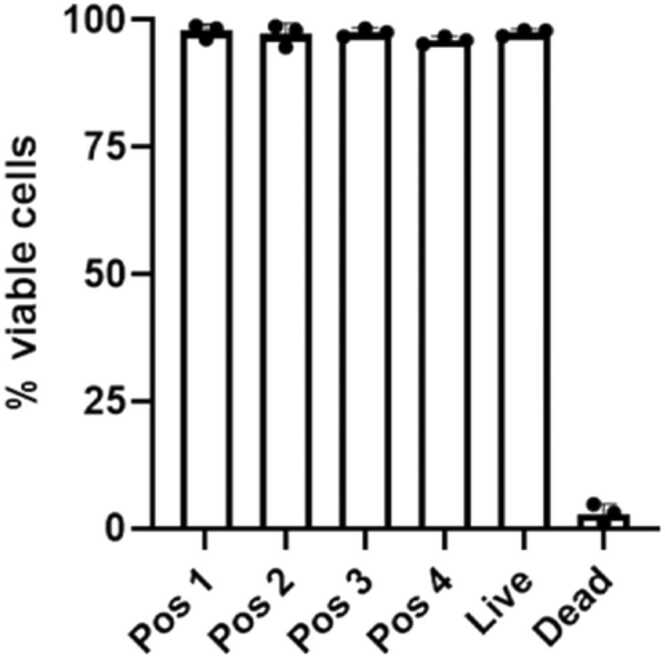
Longer-term cell survival inside NAVETTA. Viability data determined by Fixable Viability Dye eFluor506 using flow cytometry show > 95 % viable cells after 24 h exposure to clean air inside NAVETTA (all 4 positions) conditioned at ∼98 % rH and 37°C.

Long-term exposure scenarios require measurements over up to 24 h. As the NAVETTA had been designed to satisfy short-term as well as longer-term needs, A549 cells were exposed to CA (0.2 Lpm) inside the NAVETTA for 24 h. Viability determined by flow cytometry analysis (Fig. 3) resulted in values > 95 %. This was confirmed by MTT cell viability assay showing a viability of 98 % and 89 % for 0.2 and 0.3 Lpm, respectively. Thus, cell survival at typical operating conditions and airflow rates allows addressing long-term exposure scenarios.

### 3.3 Reproducibility studies

First, the reproducibility of the NAVETTA system for deposition of materials delivered *via* the air was tested for 3–4 independent biological repeats and the run-to-run variability was determined. A set of different aerosols generated either from aqueous solution (fluorescein-sodium salt as detection reagent) and particles from PBS suspension (pristine FITC-labeled silica NP) were used. The goals for reproducibility were set at a run-to-run and a position-to-position coefficient of variation (CV%) of < 15.

#### 3.3.1 Aerosolizing fluorescein droplets from aqueous solution show satisfying run-to-run reproducibility and low position-to-position variability

**Fig. 4:**
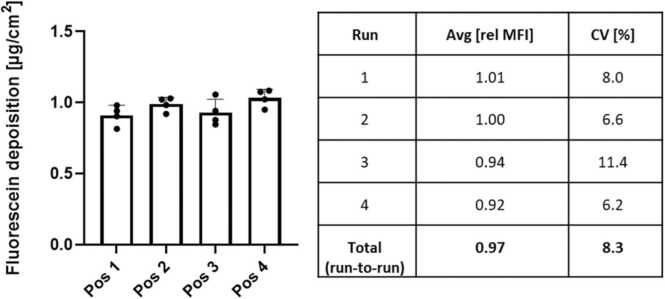
Run-to-run and position-to-position variability for fluorescein droplets. The NAVETTA system shows reproducible run-to-run deposition as well as a low position-to-position variability. Fluorescein (0.1 %) aerosolized by collision-type atomizer. The table to the right lists the average fluorescein deposition and the coefficients of variation (CV%) representing the position-to-position variability within one run and over four runs.

Cells were exposed to the well-characterized fluorescein droplets (Fig. 2A) and the deposited concentration was determined by measuring the fluorescence (n = 4 per run or per position). Fig. 4 shows the fluorescein concentrations and standard deviations (absolute) per position and run. The graph depicts a homogeneous deposition of the fluorescein across all four positions with a CV% of 5–10, while CV% runs were varying between 6.2 and 11.4.

#### 3.3.2 Pristine FITC-labeled silica NP can be charged and deposited under different conditions at low run-to-run and spatial variability

**Fig. 5:**
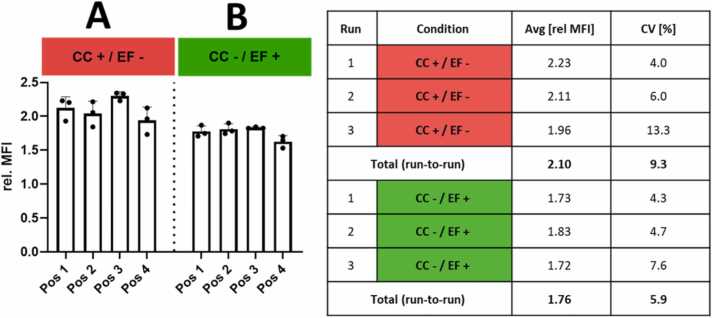
Run-to-run and position-to-position variability for pristine FITC-labeled silica NP. The NAVETTA system shows reproducible run-to-run deposition as well as a low position-to-position variability. A. Deposition, measured as mean fluorescence intensity (MFI), of additionally positively charged aerosol (using a corona charger, CC+) in a negative electric field (EF-). B. Deposition, measured as MFI, of additionally negatively charged (CC-) aerosol in a positive EF (EF+). The table to the right lists average relative MFI values (relative to untreated controls) and the coefficients of variation (CV%) representing the position-to-position variability within one run and over three runs.

The run-to-run as well as position-to-position variability of the NAVETTA system on pristine (non-functionalized) FITC-labeled silica NP was determined under different conditions. The pristine FITC-labeled silica NP were monodisperse 100 ± 2 nm in size and had a negative zeta potential (-44 ± 6 mV), as reported by the manufacturer. Additionally, charged aerosol (using the CC) was deposited in the opposite EF. Those two conditions were applied for three independent runs. Deposition condition A (Fig. 5A) resulted in a higher relative mean fluorescent intensity (MFI) compared to condition B (Fig. 5B), with a run-to-run variability of 2.10 and a coefficient of variation (CV%) of 9.3 for condition A, whereas condition B resulted in slightly lower but more consistent deposition with a run-to-run variability from 1.76 and a CV% of 5.9. To allow for a comparison between conditions, data generated using the CC was adjusted using a correction factor (x / 0.7 Lpm * 0.85 Lpm) since the aerosol airstream encounters an additional dilution when entering the CC. As satisfying reproducibility across the four positions and several runs (CV% <15, n = 3) was obtained, a use of the four NAVETTA positions as individual biological replicates can be herewith proposed.

### 3.4 Versatility studies for deposition under various run conditions

Cells were exposed to aerosolized silica NP. The efficiency of the EF was tested in combination with different charging conditions of the aerosol. Additionally, surface-modified silica NP were used to study the influence of particle characteristics on electrodeposition. Finally, two dry powder generators were tested to study a pro-inflammatory response of nanoTiO_2_.

#### 3.4.1 The electrostatic field inside the NAVETTA enables effective deposition

**Fig. 6:**
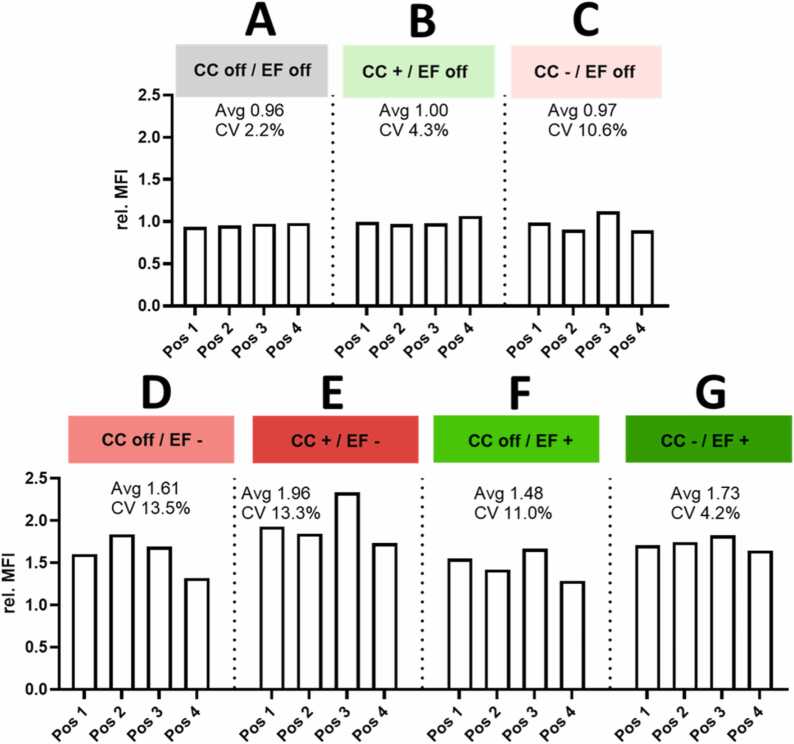
Electric field enabled deposition of intrinsic and charged aerosol. Deposition of pristine silica NP measured as mean intensity fluorescence (MFI) A-C. Deposition of not (A), positive (B) and negative (C) charged aerosol in the absence of the electric field (EF off) D. Deposition of not additionally charged particles (CC off) in a negative electric field (EF-) E. Deposition of additionally positive charged particles (CC+) in a negative electric field (EF-) F. Deposition of not additionally charged particles (CC off) in a positive electric field (EF+) G. Deposition of additionally negative charged particles (CC-) in a positive electric field (EF+).

To examine the functionality and effectiveness of the EF applied, the deposition of positively, negatively, and non-charged pristine fluorescently-labeled silica NP onto cells was determined for different run conditions without EF using flow cytometry readouts. In the absence of an EF (EF off) shown in Fig. 6A-C, three distinct scenarios were investigated. Independent of the charging inside the CC (+1 kV, −1 kV, switched off), average MFI values of approximately 1.0 were determined, indicating that aerosols were not deposited.

#### 3.4.2 The NAVETTA can be operated with aerosols using intrinsic charges in combination with the electric field

The effects of the negative intrinsic charge of pristine silica NP (-40 mV) were tested under both negative (Fig. 6D) and positive (Fig. 6F) EF. The polarity of the EF did not affect the measured MFI. However, deposition within the positive EF demonstrated more consistent position-to-position variability, with a lower CV of 11 %, compared to 13.5 % observed in the negative field. The lowest position-to-position variability of 4.2 % was achieved when pristine silica NP that were additionally negatively charged and positively deposited in the EF (Fig. 6G).

#### 3.4.3 Deposition with pristine FITC-labeled silica NP and flow cytometry readout using the four positions as bio-replicates and corona charger functionality testing with silica NP

Additional charging of the pristine silica NP in the CC resulted in an enhanced MFI for both charge options, CC positive (Fig. 6E) and CC negative (Fig. 6G) compared to the deposition achieved based on intrinsic charges. When pristine particles, which intrinsically carry negative charge, were subjected to positive charging in the CC and deposited in a negative EF, the deposition became uneven, resulting in a CV of 13.3 % between different positions.

#### 3.4.4 Surface modification affects aerosol deposition

**Fig. 7:**
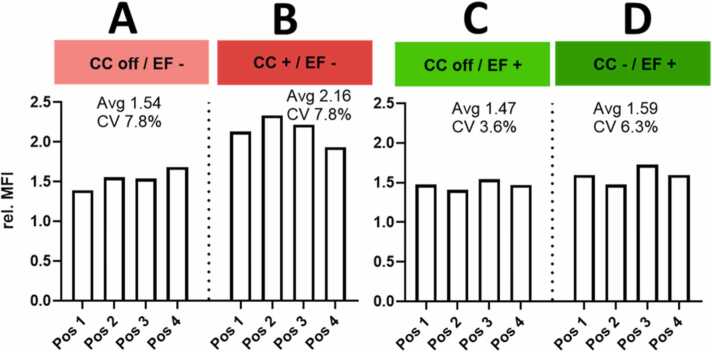
Deposition efficiency of surface-functionalized silica NP. A. Silica NP with amino surface modification without additional charging (CC off) deposited in a negative electric field (EF-); B. Silica NP with amino surface modification additionally positively charged (CC+) and deposited in a negative electric field (EF-); C. Silica NP with carboxy surface modification without additional charging (CC off) deposited in a positive electric field (EF+); D. Silica NP with carboxy surface modification with additional negative (CC-) charging deposited in a positive electric field (EF+).

Surface modifications can change the charge of the particles and, therefore, their deposition behavior. To investigate these differences, FITC-labeled silica NP with an amino group surface modification and FITC-labeled silica NP with a carboxyl group surface modification were used. Both batches were monodisperse with 100 ± 4 nm in size. The zeta potential for the amino silica NP was positive (+20 ± 4 mV) and for the carboxyl silica NP negative (-40 ± 5 mV). Two runs were performed using the positive amino silica NP, not additionally charged NP deposited negatively (Fig. 7A) and additionally positively charged aerosol deposited negatively (Fig. 7B). Two runs were performed using the negative carboxyl silica NP, not additionally charged silica NP deposited positively (Fig. 7C), and additionally negatively charged aerosol (Fig. 7D) deposited positively. Additional positive charging of amino silica NP resulted in an enhanced MFI compared to deposition achieved based on the intrinsic charge. The position-to-position variability was very similar comparing additionally positively charged particles (CV 7.8 %) and intrinsically charged (7.8 %). No difference in MFI was found comparing the additionally negatively charged particles and intrinsically charged NP carrying carboxyl functionalization. A most homogenous deposition (CV 3.6 %) occurred without additional charging of carboxy-functionalized NP in a positive EF.

#### 3.4.5 Proof-of-concept studies applying dry powder aerosolization in correlation to *in vivo* data on the inflammatory potential of inhaled TiO_2_ NP *in vitro*

*In vivo* data as well as results derived from submerged culture conditions and upon aerosol ALI exposure [21] were available from previous studies, hence, the applicability of the NAVETTA was compared using a nanoTiO_2_ reference material. Two different dry powder aerosolizer types (PI and PX) were tested to exclude any instrumental bias by the aerosolization procedure. Depending on the desired dose, cells were exposed to 1, 3 or 5 generation cycles of 1 mg each, conducting three independent runs per experiment. The TiO_2_ doses delivered onto the cells were determined using inductively coupled plasma-mass spectrometry (ICP-MS). For the PI system, the doses were 3.2 ± 1.3 µg/cm² for 1 cycle, 7.2 ± 3.9 µg/cm² for 3 cycles, and 11.5 ± 5.9 µg/cm² for 5 cycles. For the PX system, the doses were 3.2 ± 1.3 µg/cm² for 1 cycle, 9.3 ± 4.3 µg/cm² for 3 cycles, and 18.2 ± 9.2 µg/cm² for 5 cycles, respectively. Hence, 0.1–0.2 % of the dry powder material was deposited onto each transwell. To gain more insight into run-to-run and position-to-position variability for dry powders, a separate set of deposition experiments was performed for the highest dose of nanoTiO_2_ (5 ×1 mg) using the PI system, with six individual runs on one day, *i.e.* 3 runs for ICP-MS analysis and 3 runs for biological read-outs. The same settings were used as the initial experiment. The average run-to-run CV was 33 ± 8.9 % and the average position-to-position CV was 14.5 ± 9.5 %. One value was 1.7-times higher than the average deposited dose over all positions. Because of the limited dataset it was not possible to define this value as an outlier. When excluding this value, the average run-to-run CV was 29.3 ± 2.7 % and the average position-to-position CV was 11.4 ± 3.6 %.

After exposure, cells were post-incubated in the cell incubator for 19–24 h at the ALI. The controls included a negative control, which consisted of an incubator control (IC), which consisted of cells kept in a humidified incubator for the entire assay, and a cell exposure to 5 cycles of CA. An extra positive control was included to monitor IL-8 cyto-/chemokine release upon 20 μg/mL LPS. Notably, the dry aerosol nanoTiO_2_ particles were controlled for endotoxin content using a kinetic chromogenic LAL assay, which measures the incremental increase in absorbance over 150 sec. The determined endotoxin concentration was below threshold for the TiO_2_ samples, which were diluted to concentrations where the opacity of nanoTiO_2_ did not interfere with the photometric readout.

**Fig. 8:**
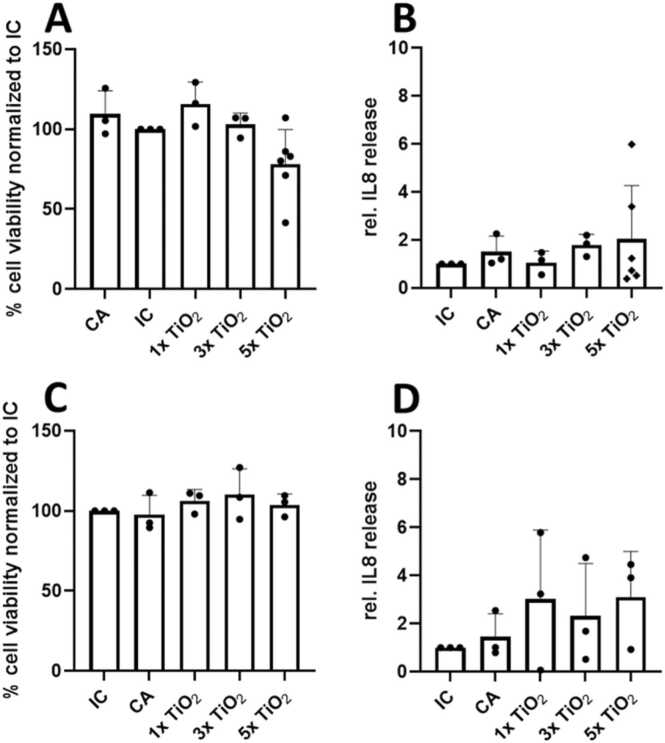
*In vitro* immune activation upon nanoTiO_2_ aerosol deposition onto ALI-cultured A549 cells. A+C. Percentage cell viability (MTT readout after 19–24 h) normalized to incubator control (IC) (=100 % cell viability) upon generation of dry powder nanoTiO_2_ aerosol after 1, 3, and 5 generation cycles (1 mg/cycle) using the PreciseInhale® (PI, A, n = 3 for 1x and 3x; n = 6 for 5x) and the PowderX (PX, C, n = 3) system. B+D. Relative IL-8 response of A549 cells exposed to clean air (CA) and dry powder nanoTiO_2_ aerosol generation compared to IC using the PI (B, n = 3 for 1x and 3x; n = 6 for 5x) and the PX (C, n = 3) system. Samples from the basolateral medium were collected for assessment of the pro-inflammatory marker after 19–24 h.

Cell viability was measured after exposure to TiO_2_ NP generated by the PI (Fig. 8A) and PX (Fig. 8C) systems, respectively. The data presented in Fig. 8A demonstrated a concentration-dependent decrease in cell survival after 5 generation cycles of nanoTiO_2_ exposure. Viability values exceeding 100 % were observed after CA, 1, and 3 generation cycles exposures, likely due to cellular stress. MTT assays measure metabolic activity, and stressed cells typically react with an enhanced metabolism, leading to elevated viability readings [26], [27]. Cell viability remained consistently high after exposure to nanoTiO_2_ generated by the PX system. The relative IL-8 secretion, illustrated in Fig. 8B (PI) and 8D (PX), showed a slight increase after CA exposure, likely caused by slightly elevated cell stress under the horizontal airflow. An elevated response was already evident after one generation cycle using the PX system, whereas this effect was less pronounced with the PI system. However, variability in IL-8 release was observed across the different runs, becoming apparent after 5 cycles with the PI system and consistently across all cycles with the PX system. Additional work is required for the discontinuously operating dry powder aerosolizers.

## 4 Discussion

This study intended to demonstrate the reproducibility and versatility of the NAVETTA system across various aerosolization conditions and particle types. The findings underscore the system’s ability to generate aerosols within the respirable size range and provide reproducible results for aerosol deposition across multiple runs and exposure conditions, making it a robust *in vitro* tool for longer-term experimental *in vitro* exposure conditions (up to 24 h), diverse materials, surface chemistries, and applications. Particle size of aerosol matters because it determines the inhalation depth. Particulate matter (PM) describes particle sizes in ambient air. PM_10_ defines particles < 10 µm in aerodynamic diameter mainly depositing in the upper airways. Especially PM_2.5_ (<2.5 µm in diameter) is able to penetrate into the deep parts of the lung. Particles in this size class are the fifth leading risk factor for death in the world, responsible for 4.2 million deaths (7.6 % of total global deaths) in 2015. PM_0.1_ (<0.1 µm in diameter), which represents the genuine nano-scale aerosol size range, can penetrate the thin epithelial barrier of the alveoli, *i.e.* 0.1–0.2 µm, and cause systemic effects. Exposure to polluted air can cause pro-inflammatory signaling causing acute inflammation, however, upon repeated exposure to persistent materials it can also cause chronic diseases [28], [29]. The particle size distributions generated by the BLAM, collision-type atomizer, and the PI and PX dry powder aerosolizer systems, used with the NAVETTA system, fall within the most harmful size class, PM_2.5_. Studying the effects of these particles on A549 cells, a model of type II alveolar epithelial cells, is appropriate for investigating their impact on the alveoli. By adjusting the aerosol size and using different cell models, the NAVETTA system can be adapted to study the effects of larger particles in other regions of the lung, increasing its versatility. Such setups were, however, beyond the scope of this study. The longer-term cell viability results indicate that the NAVETTA system provides ideal conditions for prolonged *in vitro* exposure experiments, with more than 95 % of A549 cells surviving after 24 h of exposure. This is a significant achievement for toxicology studies that require extended exposure periods to replicate real-life inhalation scenarios, *e.g.*, 8 h exposure periods for occupational settings. The ability to maintain high cell viability under the system's-controlled environment, *i.e.* 98 % relative humidity and 37°C, demonstrates its effectiveness in supporting biologically relevant conditions. The system ensures that cells can withstand the low laminar lateral airflow provided within the NAVETTA. The materials in this study were selected considering their fitness for the intended purpose based on the materials’ genuine physicochemical properties. Sodium fluorescein is widely used in deposition studies due to its ease of handling and its established role as a fluorescent tracer. It provides high detectability, making it a reliable choice for tracking and quantifying particle deposition. Fluorescent silica NP were chosen, because of their uniform size and stable labeling, making them well-traceable at the single cell level using flow cytometry. Besides, silica is among the most produced nanomaterials, making it relevant for real-world applications. Additionally, the ability to chemically modify the surface of silica NP allows for various functionalization studies, enabling to understand how surface characteristics such as charge impact deposition behavior. One of the key strengths of the NAVETTA, as highlighted by this study, is its reproducibility when operated with continuous aerosolizers opening the capacity of running several biological replicates in one go. Investigating both, fluorescein droplets and fluorescent silica NP deposition resulting in CV% < 15, suggests that the system can be reliably used for reproducible deposition experiments where the four positions can be used as biological repeats. Because run-to run variability exceeded this threshold (CV was 33 %), for dry powder further optimization work will be needed to enable reliable experimentation also with discontinuously operating aerosolizers, such as the two systems tested here. As possible explanations for these observations several options exist. On the one hand, the reproducibility of the PI system independent of deposition inside the NAVETTA was not determined in this study, which might explain the found variability. On the other hand, the removal of the membrane for the subsequent ICP-MS analyses by cutting is a manual handling compromise taken here, which might have introduced variability. This may be optimized by trypsinization of the cells from the membrane which will be included for follow-up experiments. The NAVETTA differs from other available systems in several crucial features. Electrostatic Aerosol *in Vitro* Exposure System, shortly termed EAVES, utilizes a parabolic deposition flow pattern where aerosol is delivered from above, ensuring uniform deposition on the substrate. This system employs a CC to create an EF, enhancing deposition through both electrostatic attraction and gravitational sedimentation. Notably, operating the CC at 1.5 kV produces ozone, but testing with A549 cells confirmed no immune reactions, indicating that observed IL-8 increases after TiO₂ exposure are not attributable to ozone. Deposition efficiency was quantitatively assessed using PSL fluorescent particles, achieving a total collection efficiency of 47.0 ± 9.8 % and deposition rates of 2.0 ± 0.5 % per cell unit for 0.2 µm particles [30], [31]. The Gillings Sampler is a mobile device utilizing a horizontal flow pattern to deposit aerosol onto cells in wells positioned at the bottom of the chamber. The aerosol is charged, humidified, and drawn down onto the cells through electrostatic deposition at a flow rate of 2.2 Lpm. The particle deposition efficiency was determined to be 45 % for the 6-well deposition plate (CV = 24.5 %) and 38 % (CV = 28.7 %), demonstrating consistent performance. Importantly, the device can operate for up to 4 h with high voltages active without causing adverse effects on A549 cells, as validated by IL-8 and lactate dehydrogenase endpoint measurements [32]. The ALICE Cloud exposure system is a widely used platform that enables aerosol deposition through cloud sedimentation. Using sodium fluorescein as a tracer, the deposition efficiency in a 6-well plate was evaluated, with an average of 5.6 μl of solution deposited per insert, forming a 13.3-μm thin liquid film. The system demonstrated excellent uniformity, with a mean insert-to-insert dose variability of 4.3 %. A549 cells were employed for this proof-of-concept study, highlighting the system’s reliability and precision in achieving consistent deposition [33]. In contrast to the above-mentioned systems, the NAVETTA employs a low laminar lateral airflow (0.2 Lpm) for aerosol deposition, which, while more challenging to implement compared to the commonly used parabolic or cloud sedimentation deposition, achieves comparable performance in terms of deposition uniformity and variability. A major advantage of the low CV of deposition efficiency within the NAVETTA is that it allows for four different readouts to be obtained from a single exposure run, minimizing the use of resources. This is particularly important when working with pharmaceutical materials, which are often costly and limited in supply. By reducing resource consumption, this approach enhances experimental efficiency while still ensuring that data remains reliable and precise. Another unique feature is the EF-enhanced deposition where the EF inside the NAVETTA enables deposition of additionally charged or intrinsically charged particles. To enable more homogeneous and enhanced deposition when using the EF, the aerosol can be pre-charged using a CC. In this process, ionization of CA occurs around a corona wire, creating a unipolar ion cloud. When the sample air passes through this cloud, the aerosol particles undergo diffusion charging. This happens due to the random thermal motion of ions, which collide with the aerosol particles and attach, imparting a charge to them. The polarity of the charge, whether negative or positive, depends on the EF applied at the corona wire [34], [35]. The charging of aerosols ensures a more uniform EF-mediated deposition, improving the accuracy and consistency of particle delivery to the cells. The decision regarding which charge to apply to particles can be based on NP intrinsic charge, as applying an additional charge of the same polarity can lead to more homogeneous deposition. This study also demonstrates that charging intrinsically negatively charged pristine silica NP positively, by employing a CC, results in a deposition with a position-to-position variability < 15 %. This parameter can be precisely controlled and adjusted within the NAVETTA system, allowing for customization based on the specific requirements of the experiment in scope. Deposition of not additionally charged silica NP was observed to be slightly reduced. Pristine silica NP displaying a negative zeta potential were deposited by a positive EF. In such cases deposition occurs primarily due to the intrinsic charge of the particles. However, pristine silica NP could be deposited also in a negative EF. This can be explained by the fact that particles often acquire a charge during the aerosolization process, resulting in a charged aerosol at Boltzmann equilibrium. In this case, particles with different charges are formed, and the fraction that possesses the charge opposite to the polarity of the applied EF is attracted and deposited onto the cells [36]. NanoTiO_2_ is widely used by industry, making it a relevant particulate material for inhalation exposure studies. The nanoTiO_2_ case was selected here because data from the NANoREG project was available for benchmarking. In NANoREG (FP7, Grant agreement ID: 310584) NanoTiO_2_ (NM-105) has been studied for pulmonary toxicity in rats and *in vitro* [21], [37], [38], [39] using A549 cells. After a single intratracheal exposure at 5, 50, and 500 μg/lungs, rats were sacrificed after various times. The doses mentioned correspond to 0.125, 0.0125, and 0.00125 μg/cm² assuming 4000 cm² for total rat lung alveolar surface. NanoTiO_2_ was detected in the tracheobronchial lymph nodes after 35 and 90 days. There was no significant systemic distribution in liver, kidneys, and spleen. The cytokine measurements in bronchoalveolar lavage fluid confirmed the pro-inflammatory potential of nanoTiO_2_. In *in vitro* studies, A549 cells were directly exposed to nanoTiO_2_ aerosol at the ALI (different settings were used). A maximum dose of 18 μg/cm² was achieved. Biomarkers of inflammation and cytotoxicity were assessed. No responses were observed up to a dose of 3 μg/cm². Based on these *in vivo* and *in vitro* findings, cell viability (MTT) and the pro-inflammatory marker IL-8 was assessed in this study. The aim was to reach a deposited dose higher than 3 μg/cm² nanoTiO_2_ on A549 cells in the NAVETTA exposure chamber and to observe a biological response, which was readily achieved by both dry powder generators. Furthermore, cells showed a trend of decreased viability and increased IL-8 secretion upon nanoTiO_2_ deposition, indicating potential for application. However, additional work is needed to optimize reproducibility when using dry aerosol generators due to their discontinuous operation mode. Moreover, while an immortalized cell line like A549 may not perfectly represent the biological responses of primary cells, the objective of this work was to develop and validate the performance of the NAVETTA. A549 cells were chosen for their reproducibility, ease of culture on transwells, and for their ability to provide robust biological signals, making them ideally suited for this proof-of-concept study. Their use enabled reliable replication of experiments, ensuring consistent testing of the NAVETTA system. Follow-up studies will expand on this work by employing models of primary cells to further evaluate the system's applicability under physiologically more relevant conditions. Furthermore, it should be noted that protein corona formation due to still present FBS on ALI-cultured cells may impact cell uptake kinetics and downstream biological effects, which was, however, also beyond the scope of this study.

## 5 Conclusions

This study tested the reproducibility and versatility of the market-ready *in vitro* aerosol exposure chamber NAVETTA that enables EF-enhanced deposition using various aerosolizer systems to generate aerosols from liquid solutions, particle suspensions, or dry powder. Employing continuous aerosolizers operating from solutions or particulate suspensions satisfying reproducibility across the four positions and several runs with a CV < 15 % was obtained. The four positions can, thus, be treated as individual biological replicates for resource-efficient experiments in line with the 3 R principle and ready for SSbD-guided industrial research and innovation in the advanced materials future markets. Further, the NAVETTA enables safety and pharmaceutical efficacy testing under most realistic conditions for the pulmonary route of administration. Discontinuous aerosolizers operating from dry powders evidenced feasibility for application, however, more work is needed for optimizing precision and reproducibility when pursuing in-line coupling such devices. In this proof-of-concept study the simple, robust, and frequently employed A549 cell model addressed confining biological readouts to the alveolar region. Future research will explore the application of NAVETTA for more complex biological models, such as 3D human airway epithelial models, co-culture systems or organ-on-a-chip technologies, to further mimic *in vivo* conditions and extend the NAVETTA‘s domain of application. Moreover, expanding the system’s capacity to handle a wider range of particle types, sizes, and charges will enhance its applicability in nanotoxicology and pharmacology.

## Funding

This work was funded by the EU Horizon 2020 projects NanoCommons (Grant Agreement No. 731032), NanoRIGO (Grant Agreement No. 814530), and CompSafeNano (Grant Agreement No. 101008099), the Horizon Europe project PINK (Grant Agreement No. 101137809), the SmartCERIALS project of the 10.13039/501100004955Austrian Research Promotion Agency (FFG, Grant No. 890610), and the NanoProCoV project (Grant No. CN06/2021) funded by the Austrian Agency for Education and Internationalization (OeAD).

## CRediT authorship contribution statement

**Benjamin Punz:** Writing – review & editing, Writing – original draft, Visualization, Methodology, Investigation, Formal analysis. **Magdalena Weiss:** Writing – review & editing, Writing – original draft, Methodology, Investigation, Formal analysis. **An Jacobs:** Methodology, Investigation. **Jo Van Laer:** Methodology, Investigation. **Lisa Kleon:** Visualization, Investigation. **Sylvie Remy:** Formal analysis. **Martin Himly:** Writing – review & editing, Writing – original draft, Supervision, Funding acquisition, Formal analysis, Conceptualization. **Vanessa Auer:** Visualization, Investigation. **Evelien Frijns:** Writing – review & editing, Writing – original draft, Supervision, Funding acquisition, Formal analysis, Conceptualization. **Sandra Verstraelen:** Writing – review & editing, Writing – original draft, Supervision, Funding acquisition, Formal analysis, Conceptualization.

## Declaration of Competing Interest

The authors declare to have no conflicting interests with the content of the study, have read and revised the manuscript carefully, agreed to its submission, and accepted their responsibility for the content.

## Data Availability

Raw data including relevant metadata files, photographs of the different NAVETTA setups presented, and details for the gating strategy used for flow cytometry analyses are shared to scientific community under FAIR means under CCby4–0 license at the open access platform Zenodo under this link: https://doi.org/10.5281/zenodo.13862979

## References

[1] WHO. The top 10 causes of death. [web page] World Health Organisation 2024 07.08.2024 27.09.2024 Available from: 〈https://www.who.int/news-room/fact-sheets/detail/the-top-10-causes-of-death〉.

[2] Anderson S., Atkins P., Bäckman P., Cipolla D., Clark A., Daviskas E. (2022). Inhaled medicines: past, present, and future. Pharm Rev.

[3] Metz J.K., Hittinger M., Lehr C.M. (2022). In vitro tools for orally inhaled drug products-state of the art for their application in pharmaceutical research and industry and regulatory challenges. Vitr Model.

[4] Krewski D., Andersen M.E., Tyshenko M.G., Krishnan K., Hartung T., Boekelheide K. (2020). Toxicity testing in the 21st century: progress in the past decade and future perspectives. Arch Toxicol.

[5] Russell Wms B. (1992).

[6] European-Union. DIRECTIVE 2010/63/EU OF THE EUROPEAN PARLIAMENT AND OF THE COUNCIL of 22 September 2010 on the protection of animals used for scientific purposes. Official Journal of the European Union 2010 20.10.2010 27.09.2024 Available from: 〈https://publications.europa.eu/resource/cellar/169b16db-cb47-46e3-aff5-caa8009ec295.0007.02/DOC_1〉.

[7] Gabriel M.. COMMISSION RECOMMENDATION of 8.12.2022 establishing a European assessment framework for ‘safe and sustainable by design’ chemicals and materials. European Commission 2022 08.12.2022 27.09.2024 Available from: 〈https://research-and-innovation.ec.europa.eu/system/files/2022-12/Commission%20recommendation%20-%20establishing%20a%20European%20assessment%20framework%20for%20safe%20and%20sustainable%20by%20design.PDF〉.

[8] Apel C., Kümmerer K., Sudheshwar A., Nowack B., Som C., Colin C. (2023). Safe-and-sustainable-by-design: State of the art approaches and lessons learned from value chain perspectives. Curr Opin Green Sustain Chem.

[9] Chary A., Hennen J., Klein S.G., Serchi T., Gutleb A.C., Blömeke B. (2018). Respiratory sensitization: toxicological point of view on the available assays. Arch Toxicol.

[10] Grimm D. (2019). EPA plan to end animal testing spilts scientists. Sci Total Environ.

[11] Wadman M. FDA no longer has to require animal testing for new drugs Science. 2023:127-8.American Association for the Advancement of Science 379 6628 0036-8075 27.09.2024 〈https://www.science.org/doi/epdf/10.1126/science.adg6276〉. 10.1126/science.adg6276 36634170

[12] Motta G., Gualtieri M., Bengalli R., Saibene M., Belosi F., Nicosia A. (2024). An integrated new approach methodology for inhalation risk assessment of safe and sustainable by design nanomaterials. Environ Int.

[13] Singh A.V., Romeo A., Scott K., Wagener S., Leibrock L., Laux P. (2021). Emerging technologies for in vitro inhalation toxicology. Adv Health Mater.

[14] Primavessy D., Metz J., Schnur S., Schneider M., Lehr C.M., Hittinger M. (2021). Pulmonary in vitro instruments for the replacement of animal experiments. Eur J Pharm Biopharm.

[15] Bessa M.J., Brandão F., Rosário F., Moreira L., Reis A.T., Valdiglesias V. (2023). Assessing the in vitro toxicity of airborne (nano)particles to the human respiratory system: from basic to advanced models. J Toxicol Environ Health B Crit Rev.

[16] Zavala J., Freedman A.N., Szilagyi J.T., Jaspers I., Wambaugh J.F., Higuchi M. (2020). New approach methods to evaluate health risks of air pollutants: Critical design considerations for in vitro exposure testing. Int J Environ Res Public Health.

[17] Jeannet N., Fierz M., Kalberer M., Burtscher H., Geiser M. (2015). Nano aerosol chamber for in-vitro toxicity (NACIVT) studies. Nanotoxicology.

[18] Delaval M.N., Jonsdottir H.R., Leni Z., Keller A., Brem B.T., Siegerist F. (2022). Responses of reconstituted human bronchial epithelia from normal and health-compromised donors to non-volatile particulate matter emissions from an aircraft turbofan engine. Environ Pollut.

[19] Frijns E., Verstraelen S., Stoehr L.C., Van Laer J., Jacobs A., Peters J. (2017). A Novel Exposure System Termed NAVETTA for In Vitro Laminar Flow Electrodeposition of Nanoaerosol and Evaluation of Immune Effects in Human Lung Reporter Cells. Environ Sci Technol.

[20] Gohlsch K., Mückter H., Steinritz D., Aufderheide M., Hoffmann S., Gudermann T. (2019). Exposure of 19 substances to lung A549 cells at the air liquid interface or under submerged conditions reveals high correlation between cytotoxicity in vitro and CLP classifications for acute lung toxicity. Toxicol Lett.

[21] Loret T., Rogerieux F., Trouiller B., Braun A., Egles C., Lacroix G. (2018). Predicting the in vivo pulmonary toxicity induced by acute exposure to poorly soluble nanomaterials by using advanced in vitro methods. Part Fibre Toxicol.

[22] Oostingh G.J., Schmittner M., Ehart A.K., Tischler U., Duschl A. (2008). A high-throughput screening method based on stably transformed human cells was used to determine the immunotoxic effects of fluoranthene and other PAHs. Toxicol Vitr.

[23] Rasmussen K., Mast J., De Temmerman P., Verleysen E., Waegeneers N., Van Steen F., et al. Titanium dioxide, NM-100, NM-101, NM-102, NM-103, NM-104, NM-105 – Characterisation and physico-chemical properties: Publications Office of the European Union; 2014. 〈https://data.europa.eu/doi/10.2788/79554〉.

[24] Himly M., Geppert M., Hofer S., Hofstätter N., Horejs-Höck J., Duschl A. (2020). When would immunologists consider a nanomaterial to be safe? Recommendations for planning studies on nanosafety. Small.

[25] Neun B.W., Dobrovolskaia M.A. (2011). Detection and quantitative evaluation of endotoxin contamination in nanoparticle formulations by LAL-based assays. Charact Nanopart Intend Drug Deliv.

[26] Kumar P., Nagarajan A., Uchil P.D. (2018). Analysis of cell viability by the MTT assay. Cold Spring Harb Protoc.

[27] Stoehr L.C., Endes C., Radauer-Preiml I., Boyles M.S., Casals E., Balog S. (2015). Assessment of a panel of interleukin-8 reporter lung epithelial cell lines to monitor the pro-inflammatory response following zinc oxide nanoparticle exposure under different cell culture conditions. Part Fibre Toxicol.

[28] Schraufnagel DE, Balmes J.R., Cowl C.T., De Matteis S., Jung S.H., Mortimer K. (2019). Air Pollution and Noncommunicable Diseases: A Review by the Forum of International Respiratory Societies' Environmental Committee, Part 1: The Damaging Effects of Air Pollution. Chest.

[29] Yang L., Li C., Tang X. (2020). The impact of PM2. 5 on the host defense of respiratory system. Front Cell Dev Biol.

[30] Volckens J., Dailey L., Walters G., Devlin R.B. (2009). Direct particle-to-cell deposition of coarse ambient particulate matter increases the production of inflammatory mediators from cultured human airway epithelial cells. Environ Sci Technol.

[31] De Bruijne K., Ebersviller S., Sexton K.G., Lake S., Leith D., Goodman R. (2009). Design and testing of electrostatic aerosol in vitro exposure system (EAVES): an alternative exposure system for particles. Inhal Toxicol.

[32] Zavala J., Lichtveld K., Ebersviller S., Carson J.L., Walters G.W., Jaspers I. (2014). The Gillings Sampler–An electrostatic air sampler as an alternative method for aerosol in vitro exposure studies. Chem-Biol Interact.

[33] Lenz A.-G., Stoeger T., Cei D., Schmidmeir M., Semren N., Burgstaller G. (2014). Efficient bioactive delivery of aerosolized drugs to human pulmonary epithelial cells cultured in air–liquid interface conditions. Am J Respir Cell Mol Biol.

[34] Hewitt G. (1957). The charging of small particles for electrostatic precipitation. Trans Am Inst Electr Eng, Part I: Commun Electron.

[35] Kruis F., Fissan H. (2001). Nanoparticle charging in a twin Hewitt charger. J Nanopart Res.

[36] Xi J., Si X., Longest W. (2014). Electrostatic charge effects on pharmaceutical aerosol deposition in human nasal–laryngeal airways. Pharmaceutics.

[37] Suarez-Merino B.Gd.C.F., Lacroix G.L.T., Blazy K., Peyret E.R.N., Hullo M., Broβell D.L.G., Steinborn S., Karlsson H. dB.S., Cappellini F., Prina-Mello A.M.D., di Cristo L. et al. In vitro screening methodology to evaluate toxicity by inhalation. 2016. 〈https://www.rivm.nl/sites/default/files/2019-01/NANoREG_D5_04_DR_In_vitro_screening_methodology_to_evaluate_toxicity_by_inhalation.pdf〉.

[38] Lacroix G.L.T., Rogerieux F., Protocol for inhalation exposure and choice of biological relevant endpoints. 2016. 〈https://www.rivm.nl/sites/default/files/2019-01/NANoREG_D4_15_DR_Protocol_for_inhalation_exposure_and_choice_of_biological_relevant_endpoints.pdf〉.

[39] NANoREG. NANoREG D5.04 FS In vitro screening methodology to evaluate toxicity by inhalation. 2018 Available from: 〈https://www.rivm.nl/en/documenten/nanoreg-d504-fs-in-vitro-screening-methodology-to-evaluate-toxicity-by-inhalation〉.

